# Outcomes and associated risk factors among hospitalisations attributed to invasive group A streptococcal disease in Northern Queensland, Australia: a 21-year population-based data linkage study

**DOI:** 10.1007/s15010-026-02940-6

**Published:** 2026-09-02

**Authors:** Himali Ratnayake, Damon Eisen, Oyelola Adegboye, Anton Pak, Emma McBryde

**Affiliations:** 1 College of Medicine and Dentistry, James Cook University, Townsville, QLD Australia; 2 Menzies School of Health Research, Charles Darwin University, Darwin, Northern Territory Australia; 3 Public Health and Tropical Medicine, College of Medicine and Dentistry, James Cook University, Townsville, QLD Australia; 4 Centre for Business and Economics of Health, The University of Queensland, Brisbane, QLD Australia; 5 Australian Institute of Tropical Health and Medicine, James Cook University, Townsville, QLD Australia; 6 Burnet Institute, 85 Commercial Road, Melbourne, 3004 Victoria Australia

**Keywords:** Invasive group A streptococcus, Streptococcus pyogenes, Infectious diseases, Tropical Australia, Sepsis

## Abstract

**Purpose:**

Invasive group A Streptococcus (iGAS) disease is associated with substantial morbidity and mortality. This study aimed to assess the outcomes of iGAS infection and associated risk factors in Northern Queensland, Australia.

**Methods:**

A retrospective cohort study was conducted using linked hospital data from 2000 to 2020. iGAS cases were identified using ICD-10-AM diagnosis codes in administrative health datasets. Mortality and disease burden were analysed, and risk factors for adverse outcomes were assessed using logistic regression and competing risks regression. Disability-Adjusted Life Years (DALYs) were estimated to quantify disease burden.

**Results:**

A total of 933 iGAS related hospitalisations were identified among 870 individuals. The mean age of the cohort was 45 years (SD = 24), with a median age of 61 years (IQR: 48–77 years). Hospitalised case fatality rate was 5.2%. Intensive Care Unit (ICU) admission was required for 219 patients (23.5%). Readmissions occurred in 2.0% of cases, and 3.6% experienced recurrent infection. Most deaths were attributable to sepsis (80%), with necrotising fasciitis (18%) and septic shock (13.3%) also contributing. In multivariable analysis, age ≥ 60 years was associated with lower odds of ICU admission, while female sex, non-Indigenous status, higher Socio-Economic Indexes for Areas (SEIFA) decile, cardiac disease, and renal failure were associated with increased odds of ICU admission (*p* < 0.01). Older age, non-Indigenous status and renal failure were significantly associated with increased mortality (*p* < 0.01). The total DALY burden was 1,375 years.

**Conclusion:**

iGAS imposes a substantial health burden in Northern Queensland, characterised by significant morbidity and high advanced health resource utilisation. Identification of high-risk groups highlights opportunities for targeted prevention of adverse health outcomes of iGAS.

**Supplementary Information:**

The online version contains supplementary material available at https://doi.org/10.1007/s15010-026-02940-6.

## Introduction

Group A Streptococcus (GAS) is a significant human pathogen responsible for a broad spectrum of disease, ranging from mild superficial infections to severe invasive conditions. It is recognised as one of the top 10 infectious causes of mortality worldwide [1]. Invasive GAS (iGAS) disease encompasses severe manifestations such as sepsis, bacteraemia, bacteraemic pneumonia, necrotising fasciitis (NF), streptococcal toxic shock syndrome (STSS), septic arthritis and severe skin and soft tissue infections (SSTIs). Globally, iGAS accounts for an estimated 663,000 cases and 163,000 deaths annually [2], with evidence suggesting a rising incidence in multiple regions [3, 4]. The disease contributes significantly to hospital admissions, intensive care utilisation, and healthcare costs [5]. Mortality remains clinically significant, with a notable proportion of deaths attributable to severe manifestations such as STSS [6]. Despite advances in antimicrobial therapy and appropriate supportive care, case fatality rates remain substantial even in high-income settings [5].

In Australia, recent evidence indicates an increasing burden of iGAS over time, with marked geographic and population-level disparities [6]. As of 1 July 2021, iGAS was designated a nationally notifiable disease in Australia, facilitating improved surveillance and reporting across the country [7, 8]. The incidence of iGAS varies significantly across different regions in Australia, likely influenced by regional disparities in comorbidities, socioeconomic status, and climate [9]. Notably, Indigenous people experience disproportionately higher rates of iGAS, a disparity thought to be associated with higher prevalence of chronic health conditions and social determinants such as low socio-economic status and overcrowded housing [6, 10]. The disease also appears to be more prevalent in tropical climates, suggesting a potential environmental component to transmission [6].

However, despite growing recognition of its public health importance, evidence on determinants of severe outcomes remains limited, particularly in relation to long-term population-based data. Although previous studies have primarily focused on risk factors for acquisition of iGAS, fewer have comprehensively examined predictors of disease severity and adverse clinical outcomes at a population level. This study aims to assess the adverse clinical outcomes of iGAS and identify associated risk factors using a comprehensive 21-year hospital data linkage across a region of Northern Queensland in Australia.

## Methods

### Study setting and study period

**Fig. 1:**
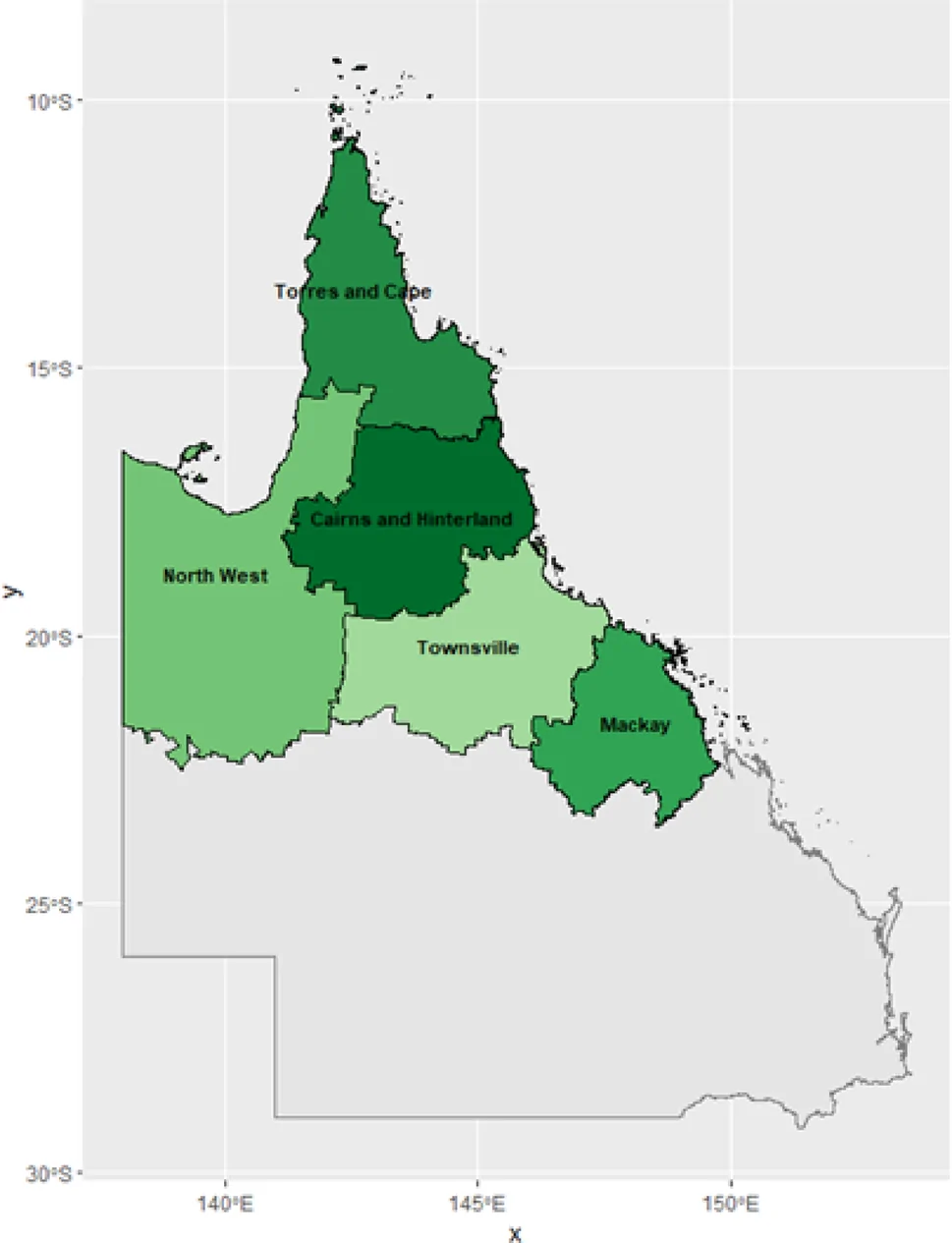
Hospital and Health Service areas included in the project

This study was conducted in five Hospital and Health Services (HHS) in Northern Queensland, namely, Cairns and Hinterland HHS, Mackay HHS, North West HHS, Torres and Cape HHS and Townsville HHS (Fig. 1). The period of data collection was from 1 January 2000 to November 2020.

### Data sources

The data for this study were obtained from a linked administrative health database comprising the Queensland Hospital Admitted Patient Data Collection, Emergency Department Collection, Death Registrations and the Australian Coordinating Registry. The linkage captures admitted patient episodes across both public and private hospitals and enables longitudinal follow-up of individuals across these collections using person-level linkage identifiers. Details of the original database, including the data sources and linkage methodology, are described by Eisen et al. [11], in which the database initially included only the Townsville Hospital and Health Service. The present study utilises an expanded version of this dataset, incorporating four additional HHSs as described above.

### Definitions

Cases of iGAS were identified using ICD-10-AM diagnosis codes (Supplementary box 1) recorded in hospital administrative data. This was a population-based data linkage study using routinely collected administrative data, and laboratory microbiology results were not available. Therefore, our case definition was based on ICD-10-AM coding for iGAS infections rather than direct microbiological confirmation. A death was classified as iGAS-related, if an iGAS-related cause was recorded as the immediate, intermediate, or underlying cause of death. Thirty-day and 7-day all-cause mortality was defined as death from any cause occurring within 30 and 7 days respectively of the index iGAS hospital admission, irrespective of whether the patient had been discharged. Mortality status was determined through linkage with the Queensland Death Registrations. A single hospitalisation episode was defined as the period from initial admission to discharge to home or usual place of residence or in-hospital death. Readmission was defined as an iGAS-related admission within 30 days of discharge from the index iGAS admission. A 30-day timeframe was selected as it is the most commonly used definition for hospital readmissions in health services research [12]. An admission occurring after 30 days of the index admission was considered a new episode.

### Statistical analysis

Data analysis was conducted using R version 4.0.3. The proportions of patients experiencing adverse outcomes, including death, ICU admission, hospital readmission, and severe disease, was calculated. Socio-demographic and clinical risk factors were assessed and presented as frequencies. The hospitalised case fatality risk was calculated as the proportion of iGAS-associated hospitalisations resulting in death during the study period. Logistic regression was used to identify determinants of ICU admission using the stats package (version 4.0.3), while a competing risks analyses were conducted using the cmprsk package (version 2.2–10) in R version 4.0.3 to examine risk factors for iGAS-related mortality, accounting for other causes of death. Cumulative incidence function (CIF) curves were generated to illustrate the cumulative incidence of death over time, stratified by cause. Disability-Adjusted Life Years (DALYs) was estimated as the sum of Years of Life Lost (YLL) and Years Lived with Disability (YLD). YLL was calculated using Australian life Tables (2010–2012) based on age-specific standard life expectancy [13], and YLD was derived by multiplying incident cases by disability weights and disease duration.

### Ethical approval

The project has obtained the ethics approval from The Townsville Hospital and Health Service Human Research Ethics Committee (HREC Reference number: HREC/16/QTHS/221) and Public Health Act approval to conduct this study. In addition, the ethics approval was obtained by the James Cook University Human Research Ethics Committee (HREC) to ensure research is conducted rigorously, respectfully, and in alignment with Indigenous rights and ethical standards.

## Results

### Cohort characteristics and outcomes

Over the 21-year study period, a total of 933 iGAS related hospitalisations were recorded among 870 patients. The mean age of the cohort was 45 years (SD = 24), with a median age of 61 years (IQR: 48–77 years). Ages ranged from less than 1 year to 93 years. There were 45 deaths attributed to iGAS, corresponding to a hospitalised case fatality rate (CFR) of 5.2%. The 7-day all-cause mortality rate was 3.4% (*n* = 30), while the 30-day all-cause mortality rate was 6.3% (*n* = 55). Of the 45 deaths, 23 (51.1%) occurred among patients admitted to the ICU. The median hospital length of stay was 8 (IQR, 4–16) days. A total of 219 patients (23.5%) required admission to an ICU while paediatric (≤ 18 years) ICU admission rate was 28.1% (43/153). The median duration of ICU care was 3 (IQR, 1–7) days and maximum being 180 days. The annual numbers of iGAS cases, incidence, and deaths over the study period are presented in Supplementary Table 1. Characteristics of iGAS-related deaths and ICU admissions are detailed in Table 1.

**Table 1:** Characteristics of deaths and ICU admissions

Characteristic	Total study population Number (%)	Deaths Number (%)	ICU Admissions Number (%)
Total number of patients	870	45	219
Sex of patient			
Male	478 (54.9)	24 (53.3)	110 (50.2)
Female	392 (45.1)	21 (46.7)	109 (49.8)
Age group (Years)			
< 1	23(2.64)	0 (0)	9 (4.1)
1–9	79 (9.1)	1 (2.2)	25 (11.4)
10–19	49 (5.6)	0 (0)	9 (4.1)
20–29	106 (12.2)	3 (6.7)	18 (8.2)
30–39	84 (9.7)	2 (4.4)	20 (9.1)
40–49	112 (12.9)	7 (15.6)	30 (13.7)
50–59	150 (17.2)	7 (15.6)	40 (18.3)
60–69	110 (12.6)	9 (20)	35 (16)
70–79	89 (10.2)	6 (13.3)	25 (11.4)
≥ 80	68 (7.8)	10 (22.2)	8 (3.7)
Indigenous Status			
Aboriginal and / or Torres Strait Islander	397(45.6)	11 (24.4)	81 (37)
Neither Aboriginal nor Torres Strait Islander	466 (53.6)	34 (75.6)	136 (62.1)
Not stated	7 (0.8)	0 (0)	2 (0.9)
Remoteness of the residence of patients			
Major Cities	0 (0)	0 (0)	0 (0)
Inner Regional	23 (2.6)	1 (2.2)	16 (7.3)
Outer Regional	573 (65.9)	35 (77.8)	138 (63)
Remote	125 (14.4)	4 (8.9)	32 (14.6)
Very Remote	149 (17.1)	5 (11.1)	33 (15.1)
Socio-Economic Indexes for Areas (SEIFA) decile*			
Most disadvantageous (1)	196 (22.5)	5 (11.1)	31 (14.2)
Relatively disadvantageous (2–4)	417 (47.9)	20 (44.4)	101 (46.1)
Averagely advantageous (5)	94 (10.8)	9 (20)	36 (16.4)
Advantageous (6–8)	145 (16.7)	11 (24.4)	46 (21)
Least disadvantageous (9–10)	18 (2.1)	0 (0)	5 (2.3)

*SEIFA deciles (1–10) were collapsed into five categories

There were 19 iGAS-related readmissions (2.04%), including six deaths during subsequent admissions, two of which were attributable to iGAS. Thirty-four individuals (3.6%) experienced recurrent iGAS infection, most of whom were male (64.7%), Indigenous (82.4%), and aged 50–59 years (38.2%). Characteristics of patients with recurrent iGAS disease is presented in Supplementary Table 2.

As shown in Table 2, sepsis was the most frequently documented clinical manifestation, occurring in 109/136 (80.1%) non-Indigenous and 59/81 (72.8%) Indigenous ICU admissions, and in 33/34 (97.1%) non-Indigenous and 10/11 (90.9%) Indigenous deaths. Necrotising fasciitis was the second commonest. Osteomyelitis was more common among Indigenous than non-Indigenous ICU admissions while streptococcal toxic shock syndrome was uncommon, occurring in 6/136 (4.4%) non-Indigenous and 3/81 (3.7%) Indigenous ICU admissions, with only 1 death.

**Table 2:** Clinical manifestations of iGAS disease among ICU admissions and deaths, stratified by Indigenous status

Clinical manifestations*	ICU admissions (*n* = 217)		Deaths (*n* = 45)	
	Non-Indigenous *N* (%)	Indigenous *N* (%)	Non-Indigenous *N* (%)	Indigenous *N* (%)
Total	136 (100.0%)	81 (100.0%)	34 (100.0%)	11 (100.0%)
Arthritis	5 (3.7%)	7 (8.6%)	2 (5.9%)	1 (9.1%)
Sepsis	109 (80.1%)	59 (72.8%)	33 (97.1%)	10 (90.9%)
Meningitis	2 (1.5%)	1 (1.2%)	1 (2.9%)	0 (0.0%)
Necrotising fasciitis	14 (10.3%)	9 (11.1%)	4 (11.8%)	3 (27.3%)
Neonatal sepsis	0 (0.0%)	1 (1.2%)	0 (0.0%)	0 (0.0%)
Osteomyelitis	4 (2.9%)	16 (19.8%)	0 (0.0%)	1 (9.1%)
Pneumonia	9 (6.6%)	1 (1.2%)	2 (5.9%)	0 (0.0%)
Puerperal sepsis	3 (2.2%)	2 (2.5%)	0 (0.0%)	1 (9.1%)
Pyothorax with fistula	2 (1.5%)	0 (0.0%)	0 (0.0%)	0 (0.0%)
Pyothorax without fistula	11 (8.1%)	2 (2.5%)	1 (2.9%)	0 (0.0%)
Retropharyngeal/ parapharyngeal abscess	7 (5.1%)	2 (2.5%)	0 (0.0%)	0 (0.0%)
Streptococcal toxic shock syndrome	6 (4.4%)	3 (3.7%)	0 (0.0%)	1 (9.1%)

* The total number of clinical manifestations does not add up to 100% as some cases were assigned more than one clinical manifestation

**Fig. 2:**
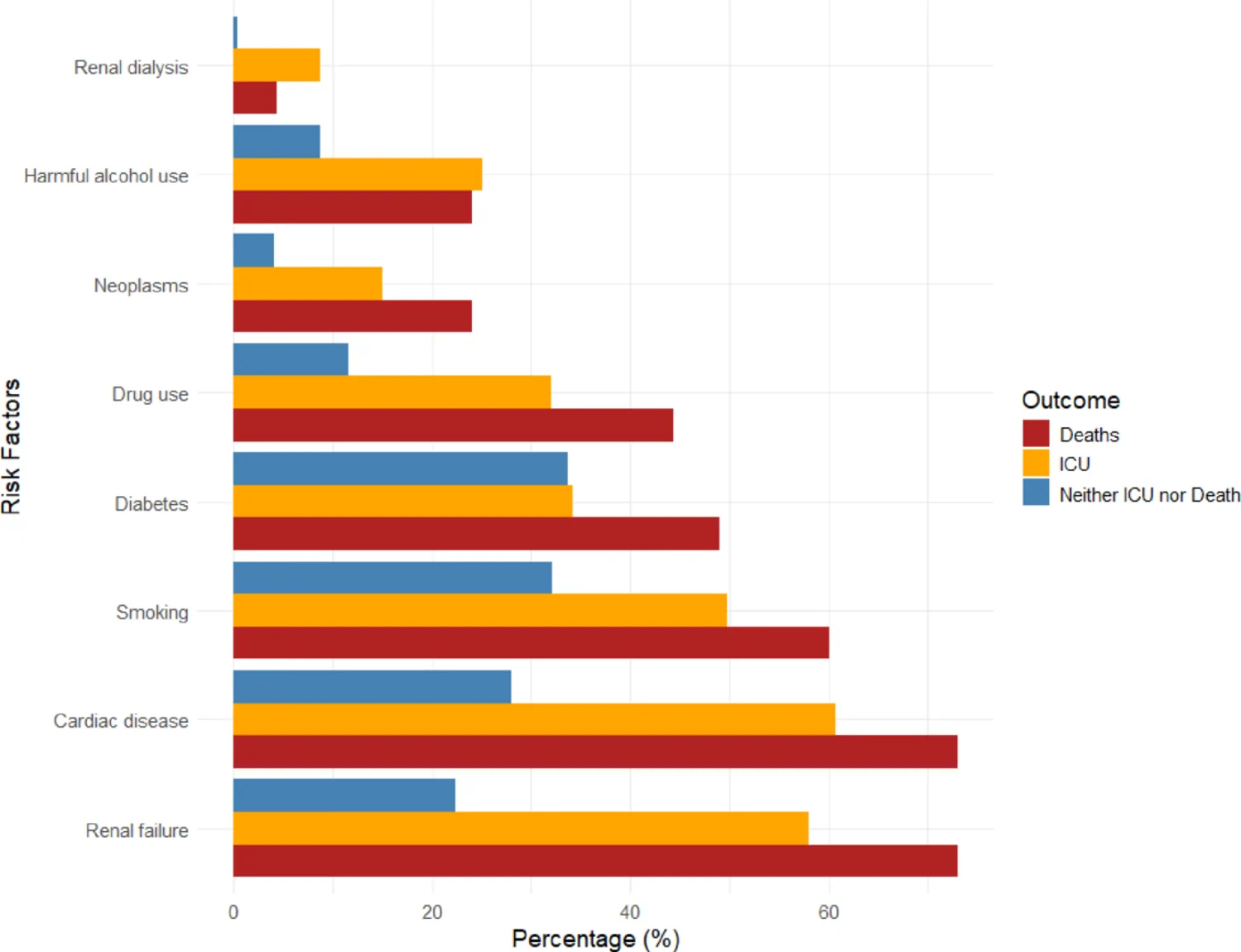
Presence of risk factors among iGAS deaths and ICU admissions compared to those who recovered without ICU admission

Distribution of known risk factors for mortality and ICU admission compared with those who recovered without ICU admission is presented in Fig. 2. Overall, both death and ICU admission were more common among patients with comorbidities and risk behaviours. Cardiac disease, renal failure, and smoking were the most frequent comorbidities observed. Cardiac disease and renal failure were present in 73% of deaths (*n* = 33) and in 60.7% (*n* = 133) and 58% (*n* = 127) of ICU admissions, respectively. Smoking was also common, occurring in 60% of deaths (*n* = 27) and 50% of ICU admissions (*n* = 109), highlighting the substantial burden of comorbidity among patients with severe outcomes. The frequency of underlying risk factors among the cases requiring ICU admission or resulting in death, stratified by Indigenous and non-Indigenous status, is presented in Supplementary Table 3.

**Fig. 3:**
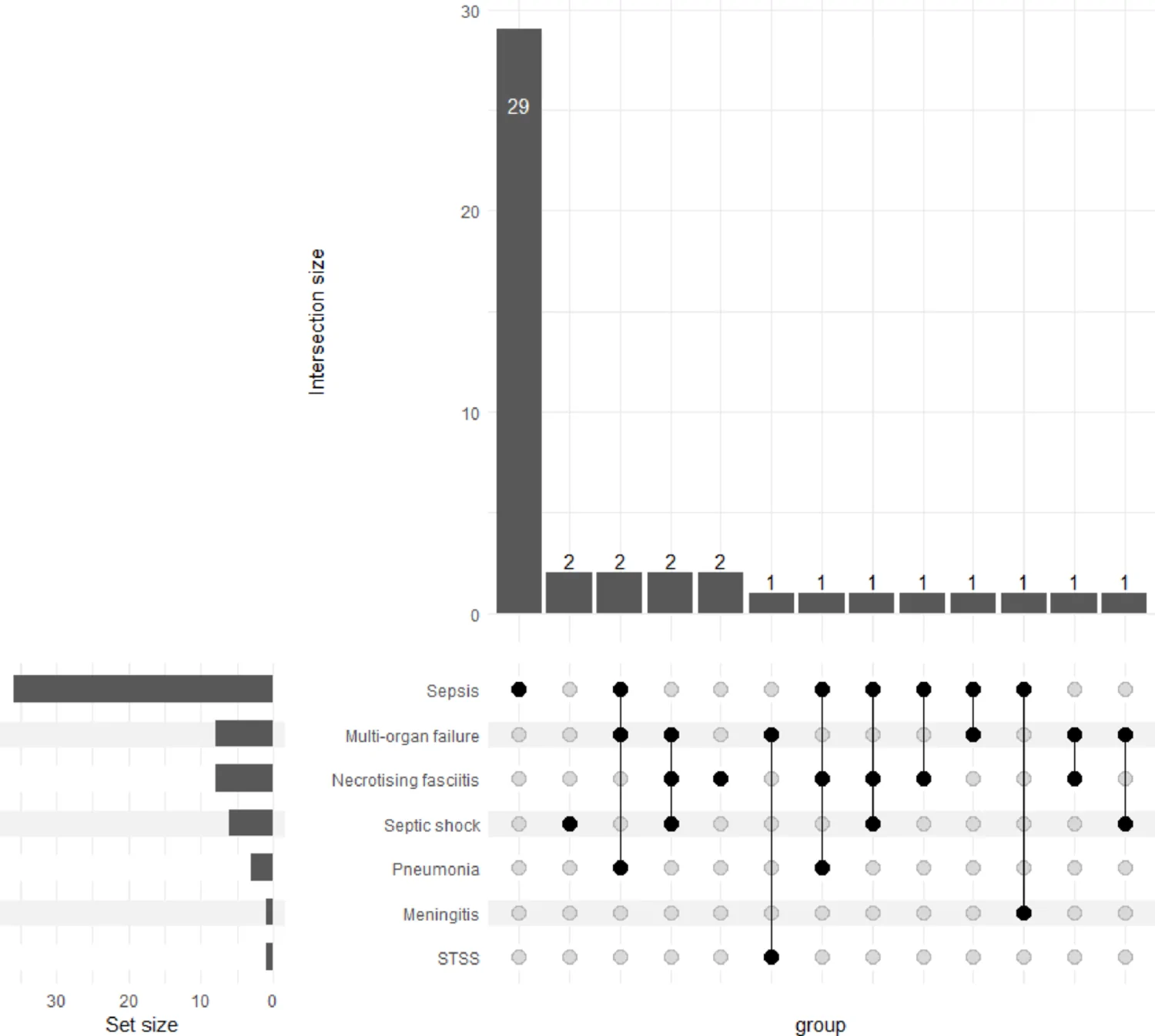
Overlapping causes of death among iGAS fatalities (*n* = 45)

We also examined the cause of death for iGAS according to the recorded diagnosis in the linked data. The majority were attributed to sepsis (*n* = 36; 80%). Additionally, 18% (*n* = 8) and 13.3% (*n* = 6) were attributed to NF and septic shock respectively. Septic shock and STSS were recorded as separate causes of death and are therefore presented as distinct categories (Fig. 3).

### Determinants of iGAS ICU admissions

Univariate and multivariable logistic regressions were performed to identify factors associated with ICU admission (Table 3). Correlation between the variables was checked by calculating the Generalised Variation Inflation Factor (GVIF). All GVIF values were well below 2, indicating no evidence of problematic multicollinearity among the predictor variables (Supplementary Table 4). In multivariable analysis, increasing age more than 60 years was associated with lower odds of ICU admission (*P* < 0.01), while female sex, non-Indigenous status, higher SEIFA decile, cardiac disease, and renal failure were associated with increased odds of ICU admission.

**Table 3:** Multivariable Logistic Regression of Factors Associated with ICU Admission

Variable	Univariate analysis		Multivariate analysis	
	Odds Ratio (OR)	*p*-value	Odds Ratio (OR)	*P*-value
Age (years)				
< 40 years	1		1	
40–59 years	1.18 (0.81–1.72)	0.38	0.79 (0.49–1.27)	0.33
≥ 60 years	1 (0.68–1.46)	0.99	0.44 (0.25–0.77)	< 0.01
Female sex	1.28 (0.94–1.75)	0.12	1.53 (1.07–2.17)	0.02
Non-Indigenous	1.70 (1.23–2.34)	< 0.01	2.26 (1.48–3.47)	< 0.01
Remoteness index*				
Regional	1		1	
Remote	0.89 (0.63–1.25)	0.49	1.31 (0.88–1.95)	0.18
SEIFA decile**	1.14 (1.07–1.23)	< 0.01	1.13 (1.04–1.23)	< 0.01
Diabetes	0.94 (0.68–1.3)	0.71	0.71 (0.45–1.13)	0.15
Cardiac disease	1.59 (1.15–2.2)	< 0.01	1.86 (1.16–2.98)	0.01
Renal failure	2.40 (1.74–3.31)	< 0.01	4.11 (2.66–6.35)	< 0.01
Renal dialysis	0.89 (0.52–1.52)	0.66	0.62 (0.33–1.16)	0.13
Harmful alcohol use	1.09 (0.76–1.55)	0.65	1.35 (0.86–2.10)	0.18
Neoplasms	1.05(0.69–1.62)	0.81	0.94 (0.58–1.53)	0.81
Smoking	0.72(0.52–0.98)	0.04	0.63 (0.38–1.03)	0.06
IV drug abuse	0.87(0.63–1.21)	0.40	0.94 (0.58–153)	0.81

*Remoteness index was categorised to two groups

**Original SEIFA decile (1 to 10) was used

### Competing risk analysis of iGAS mortality

Factors associated with iGAS-related mortality were assessed using a Fine and Gray competing risks regression model to account for the competing risk of non-iGAS deaths (Table 4). This analysis was conducted at the patient level. For individuals with multiple iGAS-associated hospitalisations, only the first (index) hospitalisation was included, and time zero was defined as the date of this admission. Subsequent readmissions or recurrent iGAS-associated hospitalisations were not included in the competing risks analysis. None of the variables showed evidence of multicollinearity, and the GVIF values for all variables were well below 2 (Supplementary Table 4). Age more than 60 years (Subdistribution Hazard Ratios (SHR) = 2.85; 95% CI: 1.59–5.11), non-Indigenous status (SHR = 2.70; 95% CI: 1.37–5.32), renal failure (SHR = 3.45; 95% CI: 1.78–6.69) and cardiac disease (SHR = 2.21; 95% CI: 1.14–4.28) were significantly associated with higher risk of death due to iGAS in univariate analysis. In the adjusted Fine and Gray model, age ≥ 60 years, non-Indigenous status, and renal failure remained independently associated with mortality, whereas cardiac disease was no longer significantly associated (*p* = 0.73).

**Table 4:** Subdistribution Hazard Ratios from Competing Risks Regression for iGAS-Related Death

Variable	Univariate analysis		Fine and Gray Model	
	Hazard Ratio (95% CI)	*P* value	Hazard Ratio (95% CI)	*P* value
Age (years)				
< 40 years	1		1	
40–59 years	1.07 (0.57–2.02)	0.82	2.78 (0.89–8.68)	0.08
≥ 60 years	2.85 (1.59–5.11)	< 0.01	3.34 (1.01–11.03)	0.048
Female sex	1.07 (0.6–1.91)	0.83	1.48 (0.78–2.8)	0.23
Non-Indigenous	2.70 (1.37–5.32)	< 0.01	2.5 (1.28–5.05)	< 0.01
Remoteness index*				
Regional	1		1	
Remote	0.55 (0.27–1.14)	0.11	0.78 (0.38–1.63)	0.51
SEIFA Decile**	1.12 (1–1.26)	0.05	1.11 (0.99–1.25)	0.07
Diabetes	1.62 (0.91–2.91)	0.1	1.26 (0.62–2.57)	0.53
Cardiac diseases	2.21 (1.14–4.28)	0.02	0.85 (0.35–2.08)	0.73
Renal failure	3.45 (1.78–6.69)	< 0.01	3.29 (1.57–6.9)	< 0.01
Renal dialysis	0.41 (0.1–1.69)	0.22	0.29 (0.07–1.29)	0.1
Neoplasms	1.59 (0.79–3.2)	0.20	0.93 (0.44–1.94)	0.84
Smoking	1.05 (0.58–1.9)	0.88	0.81 (0.34–1.92)	0.63
IV Drugs	1.42 (0.79–2.56)	0.28	1.14 (0.46–2.82)	0.77
Harmful alcohol use	0.95 (0.48–1.87)	0.88	1.18 (0.56–2.46)	0.67

*Remoteness index categorised to two groups

**Original SEIFA decile (1 to 10) was used

**Fig. 4:**
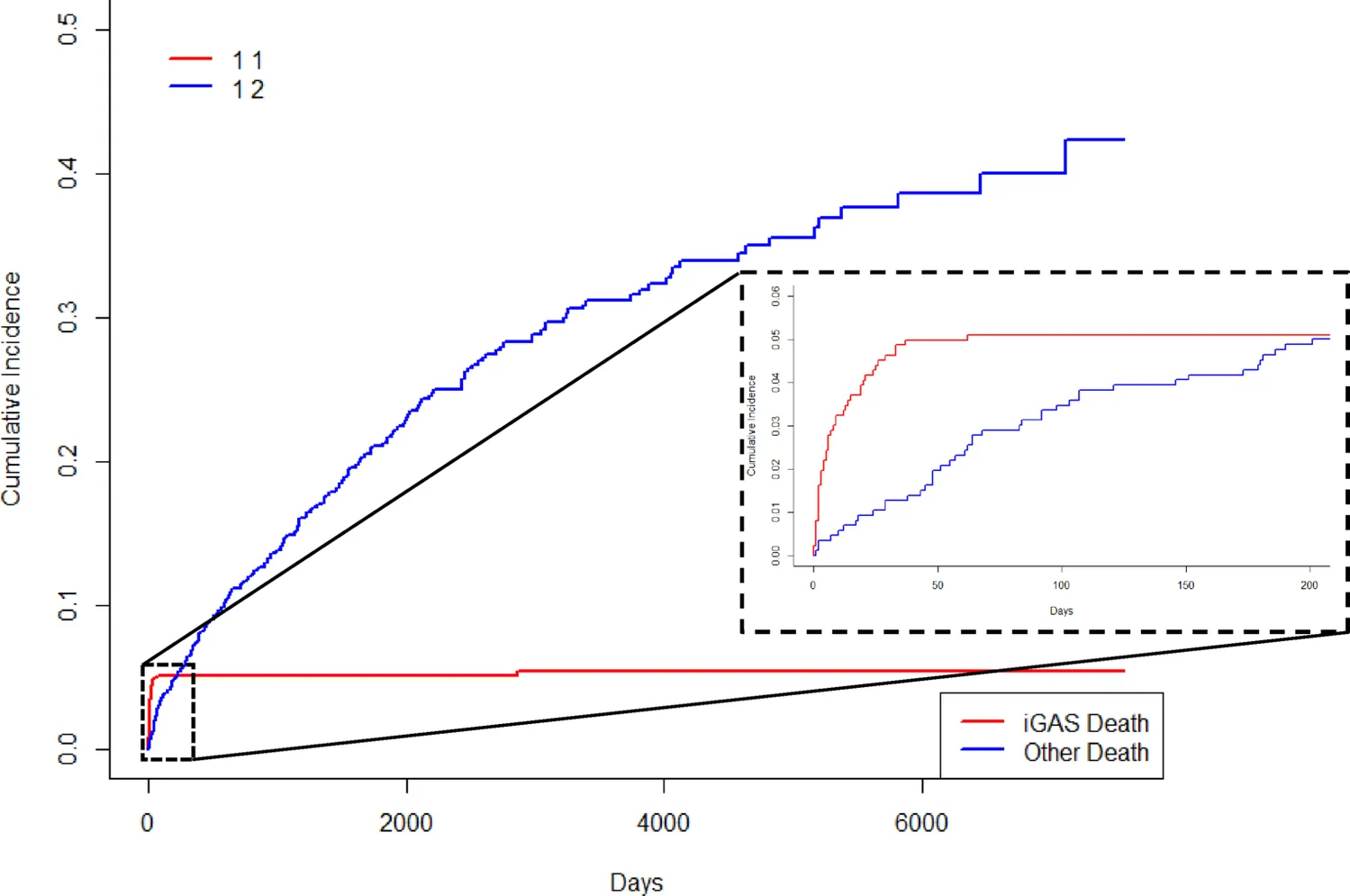
Cumulative incidence of death following iGAS, stratified by cause and over time

To visually assess the cumulative probability of iGAS-related death in comparison to deaths from other causes over time, a Cumulative Incidence Function (CIF) plot was generated (Fig. 4). The early time period was magnified to improve interpretation. The CIF plot showed that within the first 200 days following diagnosis, the probability of iGAS-related death increased more rapidly than that of other-cause mortality. Thereafter, the curves intersected, with iGAS-related mortality plateauing while deaths from other causes continued to rise, eventually reaching approximately 0.42.

### Burden of disease

YLL attributable to iGAS-related mortality was estimated at 1,370 years over the 21-year study period, reflecting a substantial burden of premature mortality in the study population. YLD was estimated at 4.7 years, calculated using a disability weight of 0.133 derived from the Global Burden of Disease (GBD) study [14]. Combining YLL and YLD yielded a total DALY burden of 1,375 years over the study period. Premature mortality accounted for approximately 99% of total health loss associated with iGAS.

## Discussion

This study demonstrates a substantial burden of severe iGAS disease in a tropical region of Australia and identifies key demographic and clinical risk factors associated with adverse outcomes. The observed CFR (5.2%) was slightly lower than previously reported rates in Australian studies (5.6% to 13.8%) [9, 15–18]. Annual mortality trends were not interpreted because the total number of deaths was small (*n* = 45), resulting in unstable annual mortality estimates. However, iGAS increased over the study period despite only a modest increase in the underlying population (Supplementary Table 1) suggesting a genuine rise in disease burden rather than changes in population size alone. The incidence of the disease is described in details in our previous paper using the same database [19]. The 7-day and 30-day all-cause mortality rates fell within the range observed in earlier studies [20–22]. Although the mortality rates are reassuring, comparisons should be interpreted cautiously due to regional differences in population characteristics.

The overall ICU admission rate in our cohort was 23.5%, which falls within the range of previously reported ICU admission rates for iGAS (6.1%–52.4%) [15, 16, 18, 20, 23] while the paediatric ICU admission rate (28.1%) was lower than more recent estimates (39.2%–46.4%) [24–27]. In contrast, ICU length of stay was longer compared to a Victorian study (median 4 days) [16]. These differences may reflect variation in disease severity as well as differences in ICU admission and discharge practices and should be interpreted cautiously given variability in clinical management across settings.

In our study, all assessed comorbidities were more prevalent among Indigenous than non-Indigenous patients. Of the comorbidities examined, renal failure was independently associated with both ICU admission and mortality, consistent with findings from a study conducted in Sydney [18]. Previous studies also reported high burden of renal failure among Indigenous patients with iGAS [9]. Despite the higher prevalence of comorbidities among Indigenous patients, non-Indigenous status remained independently associated with ICU admission and mortality after adjustment for measured covariates. While iGAS incidence is higher in Indigenous populations according to the companion study, outcomes in the non-Indigenous populations were more severe [19]. To our knowledge, this is the first Australian study to identify non-Indigenous status as an independent risk factor for both outcomes. Previous Australian studies have generally reported no significant differences in mortality between Indigenous and non-Indigenous populations [9, 20–22, 25, 28] while international findings have been inconsistent. Studies from Fiji and Alaska reported no significant differences in mortality [29, 30] whereas a Canadian study found higher case-fatality rates among non-First Nations people than First Nations people [31] and two studies from the United States reported lower mortality among non-White populations [32, 33]. The observed association may reflect factors beyond the measured comorbidities. Residual confounding may persist despite multivariable adjustment, as differences in disease severity at presentation, healthcare access, and clinical management could not be fully captured using administrative data. Consequently, this finding should be interpreted with caution and requires validation in microbiologically confirmed cohorts with detailed clinical data. Given the high burden of chronic kidney disease in Australia, these findings also highlight the importance of optimising renal health as part of comprehensive iGAS management, particularly in vulnerable populations.

Our study identified recurrent infection in 3.6% of cases (*n* = 34), compared with 4.1% (5/121) reported in a Northern Territory study [9]. In contrast to the findings of the present study, there was evidence that recurrent iGAS infection was strongly associated with patients undergoing dialysis [21]. Our study reported high proportion of renal failure, diabetes, cardiac disease and smoking among patients who experienced recurrence. Furthermore, recurrent iGAS infections were more common among Indigenous people in our analyses although non-Indigenous status was associated with more severe outcomes. One possible explanation is that Indigenous people may experience more frequent but less severe episodes, whereas non-Indigenous patients may present less frequently but with more severe disease requiring intensive care or resulting in death. This hypothesis is supported by broader health system data from the Australian Institute of Health and Welfare (AIHW) Aboriginal and Torres Strait Islander Health Performance Framework, which reported that while per-person expenditure on public hospital services for First Nations people was approximately twice that of non-Indigenous Australians, expenditure per hospitalisation was lower [34]. This may indicate that, on average, hospitalisations among Indigenous people are less resource intensive, potentially reflecting lower disease severity at presentation. However, expenditure data are an indirect proxy for disease severity, and further studies incorporating detailed clinical and microbiological data are needed to better understand the mechanisms driving these observed differences. The predominance of the 50–59-year age group among recurrent infections was an observational finding in our cohort. As our dataset did not include information on factors such as microbiological strain type, antimicrobial adherence, or other clinical characteristics that may influence recurrence, we were unable to determine the underlying reasons for this distribution.

There has been inconsistent evidence regarding the association between age and severe outcomes of iGAS in Australia [16, 22]. In our study, older age was associated with mortality, consistent with a study conducted in Victoria [16]. Similarly, two studies conducted in the United States identified increasing age as a risk factor for mortality [32, 33]. However, another study conducted in Far North Queensland found that age was not associated with death or ICU admission [22]. Differences in population characteristics, healthcare access, comorbidity profiles, and analytical approaches may contribute to the variation in findings across studies. Furthermore, the competing risks approach used in our analysis may provide a more robust estimate of attributable mortality risk by accounting for deaths from other causes. Interestingly, increasing age was significantly associated with a lower likelihood of ICU admission. This may reflect differences in ICU admission practices, comorbidity profiles, and healthcare settings across study populations.

In our study, sex was not identified as a significant risk factor for mortality; however, female sex was independently associated with ICU admission in the adjusted analysis. The reasons for this association are unclear and could not be explained by puerperal sepsis, which accounted for only 5 (2.3%) of ICU admissions. Evidence regarding the association between sex and severe iGAS disease remains inconsistent across Australian and international studies [25, 27, 28, 32, 33]. Other unmeasured factors, including differences in disease presentation, study designs, clinical management, or residual confounding, may have contributed to this finding and warrant further investigation.

Our study found higher ICU admission rates among individuals residing in areas with higher SEIFA deciles, while no significant association was observed between remoteness and ICU admission. The lack of association between remoteness and iGAS outcomes is consistent with findings from a previous Northern Territory study, which reported no significant association between remoteness and iGAS mortality [21]. One possible explanation for the higher ICU admission rates observed among individuals from higher SEIFA deciles is that these populations may have greater access to well-resourced hospitals and ICU services, potentially increasing the likelihood of ICU admission. However, further research is needed to better understand the relationship between socioeconomic status, healthcare access, healthcare utilisation patterns, and clinical outcomes.

Although strengthened by a large, linked hospital dataset and robust statistical methods, including competing risks analysis, this study has limitations. Our ICD-10-AM-based case definition differs from the Strep A Vaccine Global Consortium (SAVAC) surveillance case definition, which relies on laboratory confirmation of iGAS infection. Therefore, the accuracy of case ascertainment depends on the completeness and specificity of administrative coding, and some degree of misclassification is possible resulting in potential under- or over-ascertainment of cases. Consequently, comparisons with studies using laboratory-confirmed case definitions should be interpreted with appropriate caution. Lack of microbiological detail, including emm typing, precluded assessment of strain-specific differences in disease severity and outcomes. As the study includes only hospitalised cases, individuals who did not present to hospital or whose illness was managed in the community may not be represented. The relatively small number of deaths may have limited the statistical power and precision of the mortality analyses; therefore, these findings should be interpreted with caution. As the study period preceded the COVID-19 pandemic, the findings reflect the epidemiology of iGAS during the pre-pandemic era. Changes in respiratory virus circulation, healthcare utilisation, and the reported resurgence of iGAS following the COVID-19 pandemic may limit the generalisability of these findings to more recent populations. Nevertheless, this approach enabled the assessment of long-term outcomes and associated risk factors within a large, population-based cohort over an extended study period, providing important insights into the burden and clinical consequences of iGAS in Northern Queensland.

In conclusion, this study highlights the substantial burden of iGAS disease and identifies important factors associated with severe outcomes, including ICU admission and mortality. Although Indigenous patients experienced a higher burden of recurrent infections and comorbidities, non-Indigenous status was independently associated with increased risk of ICU admission and death after adjustment for measured covariates. These findings suggest that factors beyond underlying comorbidity profiles may influence iGAS outcomes and warrant further investigation. Future studies incorporating microbiological, clinical, and healthcare access data are needed to better understand the mechanisms underlying these disparities and inform targeted prevention and management strategies.

## Supplementary Information

Below is the link to the electronic supplementary material.

Supplementary Material 1

## Data Availability

The data underlying this study may be available from the corresponding author upon reasonable request and with appropriate approvals.
