# Supplementary material for: Outcomes and associated risk factors among hospitalisations attributed to invasive group A streptococcal disease in Northern Queensland, Australia: a 21-year population-based data linkage study
Source: Infection. 2026 Sep 2;54(5):2907–17. doi: 10.1007/s15010-026-02940-6 (PMC13727913; doi:10.1007/s15010-026-02940-6)
Supplement: Supplementary file 1 [file 15010_2026_2940_MOESM1_ESM.docx]

**Supplementary Materials**

**Supplementary Box 1: The ICD-10 codes used to identify the confirmed and probable cases of iGAS**

A 400 - Sepsis due to streptococcus, group A

B 950 - Streptococcus, group A, as the cause of diseases classified elsewhere together with one of the following codes

A 483 - Toxic shock syndrome

G002 - Streptococcal meningitis

J154 - Pneumonia due to other streptococci

J 390 - Retropharyngeal and parapharyngeal abscess

J 860 - Pyothorax with fistula

J 869 - Pyothorax without fistula

M 002 (Other streptococcal arthritis and polyarthritis) which includes M 0020 to M 0029 which further specifies the disease site

M 726 - Necrotizing fasciitis

M 86 (Osteomyelitis) which includes M 860 to M 869 which further classifies the disease type or site

M 462 to M 4628 - Osteomyelitis of vertebra

M 463 to M 4639 - Infection of intervertebral disc (pyogenic)

M 465 to M4659 - Other infective spondylopathies

O 85 - Puerperal sepsis

P 361 - Sepsis of newborn due to other and unspecified streptococci

P 3610 - Sepsis of newborn due to unspecified streptococci

P 3619 - Sepsis of newborn due to other streptococci

0 8612 - Endometritis following delivery

**Supplementary Table 1: Number of cases and deaths during the study period**

| **Year** | **Number of cases** | **Incidence per 100,000 (CI)** | **Number of deaths** |
| --- | --- | --- | --- |
| 2000 | 22 | 2.7 (1.6-3.8) | 0 |
| 2001 | 33 | 4.1 (2.7-5.5) | 2 |
| 2002 | 21 | 2.6 (1.5-3.7) | 1 |
| 2003 | 20 | 2.5 (1.4-3.5) | 2 |
| 2004 | 21 | 2.6 (1.5-3.7) | 3 |
| 2005 | 36 | 4.4 (3.0-5.9) | 2 |
| 2006 | 34 | 4.2 (2.8-5.6) | 1 |
| 2007 | 34 | 4.1 (2.7-5.5) | 1 |
| 2008 | 30 | 3.6 (2.3-4.9) | 0 |
| 2009 | 42 | 5.0 (3.5-6.5) | 4 |
| 2010 | 41 | 4.8 (3.3-6.3) | 3 |
| 2011 | 34 | 3.9 (2.6-5.2) | 3 |
| 2012 | 33 | 3.7 (2.5-5.0) | 2 |
| 2013 | 54 | 6.0 (4.4-7.6) | 7 |
| 2014 | 56 | 6.1 (4.5-7.7) | 2 |
| 2015 | 58 | 6.2 (4.6-7.8) | 2 |
| 2016 | 68 | 7.2 (5.5-8.9) | 1 |
| 2017 | 84 | 8.8 (6.9-10.7) | 5 |
| 2018 | 74 | 7.7 (6.0-9.5) | 1 |
| 2019 | 88 | 9.1 (7.2-11.0) | 3 |
| 2020 | 49 | 5.1 (3.6-6.5) | 0 |

**Supplementary Table 2: Recurrent iGAS Hospital Admissions by Patient Characteristics**

| **Variable** | **Number of repeat admissions (%)** |
| --- | --- |
| Sex | |
| Male | 22 (64.7) |
| Female | 12 (35.3) |
| Age group | |
| 1-9 | 1 (3) |
| 10-19 | 2 (5.8) |
| 20-29 | 2 (5.8) |
| 30-39 | 3 (8.8) |
| 40-49 | 4 (11.7) |
| 50-59 | 13 (38.2) |
| 60-69 | 5 (14.7) |
| 70-79 | 2 (5.8) |
| 80+ | 2 (5.8) |
| Indigenous status | |
| Indigenous | 28 (82.4) |
| Non-Indigenous | 6 (17.6) |
| Renal failure |  |
| Yes | 28 (82.4) |
| No | 6 (17.6) |
| Renal dialysis |  |
| Yes | 10 (29.4) |
| No | 24 (70.6) |
| Diabetes |  |
| Yes | 26 (74.5) |
| No | 8 (23.5) |
| Cardiac disease |  |
| Yes | 28 (82.4) |
| No | 6 (17.6) |
| Smoking |  |
| Yes | 28 (82.4) |
| No | 6 (17.6) |
| Harmful use of alcohol |  |
| Yes | 17 (50) |
| No | 17 (50) |
| Neoplasms |  |
| Yes | 5 (14.7) |
| No | 29 (85.3) |
| IV drug use |  |
| Yes | 20 (58.8) |
| No | 14 (41.2) |

**Supplementary Table 3: Frequency of underlying risk factors among Indigenous and non-Indigenous iGAS cases requiring ICU admission or resulting in death**

| **Risk factor** | **Among iGAS deaths (n=45)** | | **Among iGAS ICU admissions (n=217)** | |
| --- | --- | --- | --- | --- |
|  | **Indigenous**  **N (%)** | **Non-Indigenous**  **N (%)** | **Indigenous**  **N (%)** | **Non-Indigenous**  **N (%)** |
| **Total** | **11(100%)** | **34 (100%)** | **81 (100%)** | **136 (100%)** |
| Renal failure | 10 (90.9%) | 23 (67.6%) | 56 (74.7%) | 71 (53.8%) |
| Cardiac disease | 11 (100%) | 22 (64.7%) | 58 (77.3%) | 75 (56.8%) |
| Renal dialysis | 1 (9.1%) | 1 (2.9%) | 17 (22.7%) | 2 (1.5%) |
| Diabetes | 8 (72.7%) | 14 (41.2%) | 46 (61.3%) | 29 (22%) |
| Neoplasms | 1 (9.1%) | 9 (26.5%) | 9 (12%) | 24 (18.2%) |
| Smoking | 10 (90.9%) | 17 (50%) | 50 (66.7%) | 59 (44.7%) |
| IV Drug use | 8 (72.7%) | 12 (35.3%) | 31 (41.3%) | 39 (29.5%) |
| Harmful use of alcohol | 5 (45.5%) | 6 (17.6%) | 30 (40%) | 25 (18.9%) |

**Supplementary Table 4: Variance Inflation Factors of the variables used in logistic regression and competing risk regression**

| **Variable** | **GVIF** |
| --- | --- |
| Age group | 1.2 |
| Sex | 1.05 |
| Indigenous status | 1.21 |
| ARIA Decile group | 1.08 |
| Diabetes | 1.34 |
| Cardiac disease | 1.38 |
| Renal failure | 1.29 |
| Neoplasms | 1.07 |
| Smoking | 1.42 |
| IV Drugs | 1.36 |
| Harmful alcohol use | 1.16 |

All GVIF (Generalised Variance Inflation Factors) are well below 2.
